# Pre-Mastectomy Breast Reconstruction Intentions in Women with Breast Cancer: Psychosocial and Personality Predictors Informing Mental Health Promotion

**DOI:** 10.3390/healthcare13141761

**Published:** 2025-07-21

**Authors:** Valentini Bochtsou, Eleni I. Effraimidou, Maria Samakouri, Spyridon Plakias, Maria-Eleni Zachou, Aikaterini Arvaniti

**Affiliations:** 1 Department of Psychiatry, Faculty of Medicine, Democritus University of Thrace, 68100 Alexandroupolis, Greece; msamakou@med.duth.gr (M.S.); aarvanit@med.duth.gr (A.A.); 2 First Surgical Department, Faculty of Medicine, Democritus University of Thrace, 68100 Alexandroupolis, Greece; eeffraem@med.duth.gr; 3 Department of Physical Education and Sports, University of Thessaly, 42100 Trikala, Greece; spyros_plakias@yahoo.gr; 4 First Surgical Department, University Hospital of Alexandroupolis, 68100 Alexandroupolis, Greece; mrl.lou@hotmail.com

**Keywords:** breast reconstruction, decision-making, personality traits, psychological factors, quality of life, mastectomy, patient-centered care, mental health promotion

## Abstract

**Background/Objectives:** Despite the psychological benefits of breast reconstruction (BR) after mastectomy, uptake remains limited among women with breast cancer. This study explores psychosocial and personality predictors of BR intentions in the pre-mastectomy phase, aiming to inform strategies for mental health promotion in oncology care. **Methods:** This cross-sectional analysis used preoperative data from a longitudinal study at a university hospital in Greece. Women with primary breast cancer scheduled for mastectomy completed a battery of validated self-report measures, including the International Personality Item Big-Five Factor Markers (IPIP-BFFM), the Hospital Anxiety and Depression Scale (HADS), and the Short Form-36 Health Survey (SF-36). Demographic, clinical, and psychosocial data were also collected. Binary logistic regression was used to examine predictors of (a) BR information-seeking and (b) BR intention. **Results:** Seventy-four women participated (mean age = 61.1 years). Older age predicted lower BR intention (Exp(b) = 0.897, 95% CI: 0.829–0.970) and information-seeking (Exp(b) = 0.925, 95% CI: 0.859–0.997). Single/divorced status was associated with reduced BR information-seeking (Exp(b) = 0.053, 95% CI: 0.005–0.549). Openness to experience significantly predicted both outcomes (BR information-seeking: Exp(b) = 1.115, 95% CI: 1.028–1.209); BR intention: Exp(b) = 1.095, 95% CI: 1.016–1.181). Higher physical health-related QoL scores were associated with increased BR intention (Exp(b) = 1.039, 95% CI: 1.007–1.072), whereas higher mental health-related QoL (Exp(b) = 0.952, 95% CI: 0.912–0.994) and higher depression scores (Exp(b) = 0.797, 95% CI: 0.638–0.996) were linked to decreased BR intent. No psychological factor significantly predicted information-seeking. **Conclusions:** These findings underscore the value of psychosocial screening and personality-informed counseling prior to surgery. By identifying individuals less likely to seek information or consider BR, pre-mastectomy assessments can contribute to tailored, mental health-promoting interventions and support informed, patient-centered surgical decision-making.

## 1. Introduction

Breast reconstruction (BR) following therapeutic mastectomy is a critical component of post-mastectomy care, offering both physical and psychological benefits [1,2,3,4]. Despite substantial evidence highlighting its positive impact on body image, self-esteem, and overall quality of life, the rates of BR remain lower than expected, suggesting that multiple factors influence women’s decision-making processes regarding BR [5,6]. The complexity of this decision underscores the need to explore the interplay of a range of considerations, including medical eligibility, personal preferences, and socio-environmental influences [1,3,7]. Given the profound psychosocial impact of mastectomy, mental health promotion initiatives aimed at supporting informed surgical decision-making, including BR surgery, are essential.

Demographic characteristics such as age, marital status, education level, and socioeconomic status are well-documented determinants of BR decisions [8,9,10,11]. Research consistently indicates that younger women, those with higher socioeconomic status, and individuals having greater access to healthcare information are more inclined to pursue BR. Conversely, advanced age, financial limitations, and insufficient awareness frequently serve as barriers to BR [12,13]. Notably, despite women aged 65 and older accounting for approximately 47% of new breast cancer diagnoses, they remain significantly underrepresented among BR recipients [14]. This disparity underscores a critical need to understand the unique decision-making dynamics and support requirements of older patients within the context of oncologic and reconstructive care. Marital or relationship status has also been identified as an influential factor, with partnered women more likely to consider BR, potentially due to increased emotional support and practical assistance during treatment and recovery [13].

The decision to undergo BR is multifaceted, influenced not only by medical and demographic factors, but also by psychological characteristics and personality traits [15,16]. While previous research predominantly focused on clinical, economic, and oncological considerations, emerging evidence underscores the role of personality in shaping decision-making processes and post-surgical satisfaction [17]. Personality traits, particularly those outlined in the big five-factor model (BFFM)—neuroticism, extraversion, openness to experience, agreeableness, and conscientiousness—have been identified as key determinants of healthcare choices, emotional adaptation, and post-treatment quality of life (QoL) [16]. Studies suggest that women with higher openness to experience are more likely to actively seek information, engage in shared decision-making, and consider BR as a viable post-mastectomy option [15]. Conversely, those with high neuroticism may experience greater decisional conflict, preoperative anxiety, and postoperative dissatisfaction, impacting their likelihood of choosing BR [18]. Individuals with a more risk-averse or passive decision-making style are less likely to explore BR options [19].

Research demonstrated that personality traits not only influence BR decision-making, but also affect postoperative psychological adjustment and satisfaction with outcomes [16]. For instance, higher extroversion and agreeableness have been associated with better coping mechanisms, improved social support utilization, and greater emotional resilience post-surgery [17]. On the other hand, neuroticism has been linked to increased distress, dissatisfaction with surgical results, and a heightened perception of physical complications [18]. These findings suggest that personality assessments could be an essential tool in preoperative counseling, helping clinicians tailor their communication and support strategies to individual patient needs [15,20,21].

Additionally, psychological factors—including fear of cancer recurrence, body image concerns, and anxiety regarding additional surgeries—can shape attitudes toward BR [12,22]. Evidence suggests that women who report higher emotional well-being prior to initiating treatment may be more inclined to accept their post-mastectomy body and decline reconstructive surgery [22]. In this context, psychological functioning characterized by emotional stability, effective coping, and positive adaptation may diminish the perceived need for surgical restoration. Conversely, several studies have shown that women with heightened preoperative psychological distress—such as symptoms of depression or anxiety—are also less likely to pursue BR, potentially due to decisional fatigue, motivational impairments, or fear of complications [23]. These findings underscore the dual role of psychological health in BR decision-making: both high and low emotional functioning may lead to reconstruction refusal, albeit for different reasons. As such, incorporating systematic psychosocial assessments into preoperative care can help clinicians better understand individual motivational profiles and tailor decision support interventions accordingly [24].

Multiple studies consistently demonstrated that BR—particularly using autologous tissue—leads to improved long-term satisfaction with body image and psychosocial outcomes compared to mastectomy alone or implant-based BR [25,26]. Moreover, satisfaction appears to be sustained or even improved several years postoperatively, especially among women who underwent deep inferior epigastric perforator (DIEP) flap procedures [25]. Studies also suggest that while immediate and delayed BR both yield positive health-related QoL outcomes, immediate procedures may mitigate the psychological impact of mastectomy defects and facilitate a smoother emotional recovery [27]. These findings highlight the central role of health-related QoL in guiding shared decision-making processes between clinicians and patients considering reconstructive options.

Despite growing efforts to promote shared decision-making in oncology care, disparities in BR awareness and access persist. Misinformation, limited physician–patient discussions, and inadequate psychosocial support contribute to decisional conflict and uncertainty [6,28]. For instance, ethnic and linguistic disparities have been reported, with non-native speakers being less likely to receive adequate information about BR options [28]. Additionally, financial barriers play a crucial role, with studies showing that financial toxicity significantly affects BR decision-making, particularly among low-income women [29,30].

While sociodemographic and systemic barriers are well-documented, less is known about the intrapersonal factors—such as psychological distress, personality traits, and perceived QoL—that shape a woman’s readiness to consider BR during the preoperative period [31]. Most existing studies focus on clinical eligibility or postoperative satisfaction, often overlooking the cognitive and emotional processes involved in early decision-making [16,24]. Given that the period preceding mastectomy is characterized by heightened emotional vulnerability and time-sensitive choices, understanding the predictors of both informational desire and surgical intention is critical [22]. Such insights may inform more effective, patient-centered counseling that not only conveys medical facts, but also responds to the psychological and personality-based needs of each woman facing breast cancer surgery.

Although detailed regional epidemiological data are limited, national statistics report over 7000 new breast cancer diagnoses annually in Greece, with similar incidence patterns observed in urban and regional centers [32]. This study aims to examine the key factors shaping women’s desire for information and their intention to undergo BR following therapeutic mastectomy. Building on evidence from systematic reviews and extending it through original empirical research, this study explores how personality traits, psychological characteristics, and perceived quality of life influence Greek women’s desire for information and their preoperative decision-making regarding BR. Accordingly, we sought to assess whether specific demographic, psychological, and personality-related variables predict (a) the desire for BR-related information and (b) the intention to undergo BR among women treated at a regional academic hospital in northern Greece. Given the study‘s exploratory design and modest sample size, no a priori hypotheses were formulated. These insights may inform targeted, patient-centered interventions that facilitate informed decision-making and promote postoperative satisfaction.

## 2. Materials and Methods

### 2.1. Study Design

This article presents findings from the preoperative phase (Phase 1), prior to breast surgery, of a longitudinal, multi-phase research project investigating psychosocial determinants of BR decision-making among Greek women undergoing therapeutic mastectomy at a regional academic hospital in northern Greece. The overall study adopted a prospective, observational design and followed participants at four key time points: (Phase 1) preoperatively, (Phase 2) 4 weeks postoperatively at the onset of adjuvant therapy, (Phase 3) six months after surgery, and (Phase 4) one year postoperatively.

Although the broader project was designed as a longitudinal study, the present analysis focuses exclusively on cross-sectional data collected during the preoperative phase (Phase 1), prior to any surgical or adjuvant intervention. This design was chosen to capture participants’ baseline psychological status and initial attitudes toward BR. A cross-sectional approach was considered appropriate given the clinical and logistical constraints of the setting, where long-term follow-up was not feasible within the timeframe of the current study. As such, the findings reflect women’s preferences, intentions, and psychological functioning at a single time point, prior to undergoing mastectomy.

### 2.2. Sample

Participants were recruited between 1 October 2019 and 30 April 2023. Data collection for the preoperative phase (Phase 1) was completed during this period at the University General Hospital of Alexandroupolis. Eligible participants were women aged 18 years or older with a confirmed diagnosis of primary breast cancer, scheduled to undergo mastectomy. Additional inclusion criteria included the ability to communicate effectively in Greek and awareness of their diagnosis. Recruitment was conducted consecutively at the Breast Disease Clinic and the First Department of Surgery of the University General Hospital of Alexandroupolis.

The University General Hospital of Alexandroupolis is the primary tertiary care center for Eastern Macedonia and Thrace, a region with an estimated population of 600,000. Although formal regional cancer registry data are unavailable, national statistics report approximately 7000 new breast cancer diagnoses annually in Greece [32], suggesting an expected case load of several hundred patients per year in this region. During the study period (1 October 2019 to 30 April 2023), a total of 320 women with a confirmed diagnosis of breast cancer were assessed at the Breast Disease Unit of the First Department of Surgery, Democritus University of Thrace, at the University General Hospital of Alexandroupolis. As the main surgical referral center in the area, the hospital manages a substantial proportion of mastectomy cases within this catchment population. Thus, while the study sample is not statistically representative of all breast cancer patients in Greece, it is considered broadly reflective of the therapeutic mastectomy population treated in this regional clinical context.

To minimize selection bias, consecutive sampling was employed. The sample size for Phase 1 was determined pragmatically, based on the available patient population during the recruitment window. A formal power analysis was not conducted due to the exploratory nature of this phase.

### 2.3. Instruments

A structured battery of self-report instruments was used, including the following:

- A demographic and clinical information questionnaire, developed by the research team was used for the purposes of the study. The form collected data on age, education level, marital and parental status, professional background, religion and residential location, as well as clinical variables such as time of breast cancer diagnosis, prior surgical and oncological treatments (chemotherapy, radiotherapy, and hormone therapy), menstrual status, comorbidities, family history of breast cancer, recent exposure to stressful or traumatic events, and subjective appraisals of illness severity and perceived health threat. Two additional study-specific, closed-ended items addressing (a) the desire to receive BR-related information and (b) current BR intention, were also completed alongside the demographic and clinical questions;
- The Greek version of the 50-item International Personality Item Pool Big-Five Factor Markers (IPIP-BFFM) was used to assess personality traits, measuring five domains: extraversion, agreeableness, conscientiousness, neuroticism, and openness. Responses were rated on a 5-point Likert scale ranging from 1 (very inaccurate) to 5 (very accurate). The instrument includes both positively and negatively worded items. Prior to analysis, all negatively keyed items were reverse scored to ensure that higher scores consistently reflected a greater presence of the respective trait. The five resulting composite scores (openness, conscientiousness, extraversion, agreeableness, and neuroticism) were then entered as continuous predictors in the logistic regression models. The Greek version demonstrated good internal consistency, with Cronbach’s alpha coefficients ranging from 0.758 to 0.875 across domains [33];
- The Greek version of the Hospital Anxiety and Depression Scale (HADS), a 14-item instrument comprising two subscales (anxiety and depression) was used. Each item is scored on a 4-point Likert scale (0–3), yielding subscale scores from 0 to 21. The Greek version of the HADS has shown excellent psychometric properties, with Cronbach’s alpha values of 0.829 (anxiety) and 0.840 (depression) [34,35,36];
- The Greek version of the 36-item Short Form Health Survey (SF-36) assessing health-related quality of life was used. The tool provides eight domain scores and two composite indices: Physical Component Summary (PCS) and Mental Component Summary (MCS). The instrument includes both positively and negatively worded items. Prior to analysis, all negatively keyed items were reverse scored to ensure that higher scores consistently reflected a greater presence of the respective characteristic. Higher scores indicate better self-perceived health status. The Greek version demonstrated robust reliability (Cronbach’s α > 0.79 across all domains) and construct validity in the general population [37].

Standardized instruments and self-administration were used to reduce interviewer and measurement bias. Participants were ensured confidentiality to encourage accurate disclosure and to minimize social desirability or recall bias. All questionnaires were self-administered in a private room within the hospital. A trained clinical psychologist was present throughout the process to provide clarification and support as needed. Participants were explicitly encouraged to seek assistance with any items they did not fully understand, ensuring accurate comprehension and completion. No instances of refusal or comprehension difficulty were reported. Completion of the full battery of questionnaires, including the demographic form and validated psychometric tools, required on average of 30 min. This time frame was generally well tolerated, and no participant expressed discomfort or fatigue. The duration was deemed acceptable within the clinical setting and did not interfere with routine care procedures.

### 2.4. Ethical Approval

The study protocol was conducted in accordance with the Helsinki Declaration and ethical approval was obtained by the Ethics Committee of the University General Hospital of Alexandroupolis (Protocol code: 15 Δ.Σ, date of approval: 13 November 2019). Informed consent was obtained from all participants after they received comprehensive written information about the study. 

### 2.5. Statistical Analysis

Descriptive statistics were used to summarize the sample characteristics. For categorical variables, the absolute frequency and the percentage in each category is reported, while for scale variables, the mean and standard deviation (SD) is provided.

Binary logistic regression was used to examine associations between independent variables and two binary outcomes: (a) the desire to receive BR-related information and (b) the intention to undergo BR. A total of six binary logistic regressions were conducted to investigate the influence of demographic, personality-related, and psychological factors. All variables used in the statistical analyses are presented in Table 1. Covariates were selected based on their established relevance in prior breast reconstruction research and theoretical associations with patient decision-making. The grouping of variables reflects empirically supported domains commonly examined in psychosocial oncology and surgical decision-making. Demographic factors such as age and marital status have been consistently linked to both BR uptake and information-seeking behavior [3,13]. Personality traits—particularly openness to experience—have been implicated in greater cognitive flexibility and proactive participation in preference-sensitive healthcare decisions [18,20]. Finally, psychological constructs, including health-related QoL and depressive symptomatology, have been associated with emotional readiness and the capacity to engage in treatment planning [10,23].

The effect of demographic, personality, and psychological factors on the two dependent variables (desire for BR information, intention to undergo BR) was studied separately due to the limited sample size, ensuring that the assumptions for binary logistic regression were met. Exp(b) was reported to represent the effect size of each predictor. No multivariable model was constructed in order to preserve statistical power and model interpretability within the constraints of the available sample. The IBM SPSS Statistics software (Version 29, IBM SPSS Inc., Chicago, IL, USA) was used for statistical analysis. The significance level for all analyses was set at 0.05. Exp(b) was used to determine the effect size of each independent variable on the dependent variables.

No missing data were observed for the key variables included in the analysis. The data supporting the findings of this study are available from the corresponding author upon reasonable request.

## 3. Results

### 3.1. Descriptive Statistics

A total of 74 women participated in the survey. Of the 80 eligible patients approached, 74 consented to participate and completed the Phase 1 questionnaires (participation rate: 92.5%). One patient declined participation, one passed away during the 12-month study period, and one did not undergo breast cancer surgery but was instead placed under clinical follow-up. Additionally, three participants were later diagnosed with benign breast lesions and were excluded from the study.

The descriptive statistics (demographic, psychosocial, and clinical characteristics) for the variables used are presented in Table 1.

**Table 1:** Descriptive statistics.

Variables	Categories	Frequency	Percent	Mean	SD
Religion group	1 = Cristian	59	79.70%		
	2 = Muslim	15	20.30%		
Residence	1 = Urban area	51	68.90%		
	2 = Non-Urban area	23	31.10%		
Professional status	1 = Unemployed	17	23.00%		
	2 = Employed	25	33.80%		
	3 = Retired	32	43.20%		
Marital status	1 = Single/divorced	10	13.50%		
	2 = Widowed	22	29.70%		
	3 = Married	42	56.80%		
Age				61.14	13.95
Education level				9.19	3.84
Desire for Information	Yes	29	39.20%		
	No	45	60.80%		
Intention to undergo BR	Yes or I will think about it later	34	45.90%		
	No	40	51.10%		
IPIP_Extraversion				30.16	7.33
IPIP_Agreeableness				45.01	4.66
IPIP_Conscientiousness				41.32	5.31
IPIP_Neuroticism				29.09	9.33
IPIP_Openness				30.03	7.37
SF36_Physical health				72.93	22.34
SF36_Mental health				63.58	21.01
HADS_Anxiety				7.58	5.06
HADS_Depression				5.53	4.09

Abbreviations: BR = breast reconstruction, IPIP = International Personality Item Pool, SF36 = 36-Item Short Form Survey, HADS = Hospital Anxiety Depression Scale, and SD = standard deviation. Source: own elaboration.

#### 3.1.1. Effect of Demographic Characteristics on the Desire for BR Information

The effect of demographic characteristics (religion group, residence, professional status, marital status, age, and education level) on the desire for BR information was examined. The procedure modeled “Yes” as the response, treating “No” as the reference category. Based on the results presented in Table 2, the desire for BR information (a) decreases with increasing age (Exp(b) = 0.925, 95% CI: 0.859–0.997), and (b) decreases in the single/divorced category compared to the married category (Exp(b) = 0.053, 95% CI: 0.005–0.549).

#### 3.1.2. Effect of Demographic Characteristics on the Intention to Undergo BR

The effect of demographic characteristics (religion group, residence, professional status, marital status, age, and education level) on the intention to undergo BR was examined. The procedure modeled “Yes” or “I will think about it later” as the response, treating “No” as the reference category. Based on the results presented in Table 3, the intention to undergo BR decreases with increasing age (Exp(b) = 0.897, 95% CI: 0.829–0.970).

#### 3.1.3. Effect of Personality Characteristics on the Desire for BR Information

The effect of personality characteristics (IPIP_Extraversion, IPIP_Agreeableness, IPIP_Conscientiousness, IPIP_Neuroticism, and IPIP_Openness) on the desire for BR information was examined. The procedure modeled “Yes” as the response, treating “No” as the reference category. Based on the results presented in Table 4, the desire for BR information increases when IPIP_Openness increases (Exp(b) = 1.115, 95% CI: 1.028–1.209).

#### 3.1.4. Effect of Personality Characteristics on the Intention to Undergo BR

The effect of personality characteristics (religion group, residence, professional status, marital status, age, and education level) on the intention to undergo BR was examined. The procedure modeled “Yes” or “I will think about it later” as the response, treating “No” as the reference category. Based on the results presented in Table 5, the intention to undergo BR increases when IPIP_Openness increases (Exp(b) = 1.095, 95% CI: 1.016–1.181).

#### 3.1.5. Effect of Psychological Characteristics on the Desire for BR Information

The effect of psychological characteristics (SF36_Physical health, SF36_Mental health, HADS_Anxiety, and HADS_Depression) on the desire for BR information was examined. The procedure modeled “Yes” as the response, treating “No” as the reference category. Based on the results presented in Table 6, none of the psychological characteristics contribute statistically significantly to changes in desire for BR information.

#### 3.1.6. Effect of Psychological Characteristics on the Intention to Undergo BR

The effect of psychological characteristics (SF36_Physical health, SF36_Mental health, HADS_Anxiety, and HADS_Depression) on the intention to undergo BR was examined. The procedure modeled “Yes” or “I will think about it later” as the response, treating “No” as the reference category. Based on the results presented in Table 7, the intention to undergo BR (a) increases with increasing SF36_Physical health (Exp(b) = 1.039, 95% CI: 1.007–1.072), (b) decreases with increasing SF36_Mental health (Exp(b) = 0.952, 95% CI: 0.912–0.994), and (c) decreases with increasing HADS_Depression (Exp(b) = 0.797, 95% CI: 0.638–0.996).

### 3.2. Summary of Key Findings

Overall, the results reveal that older age consistently predicted both a reduced desire for BR-related information and a lower likelihood of intending to undergo BR. Women who were single or divorced were also significantly less likely to seek information about BR. Among personality traits, openness to experience emerged as a strong predictor of both outcomes, highlighting its role in shaping proactive health decision-making. In terms of psychological functioning, higher physical health-related quality of life was associated with increased BR intention, while higher mental health scores and depressive symptomatology were both negatively associated with BR intent. Notably, none of the psychological variables significantly predicted information-seeking behavior, suggesting that informational interest may be more closely linked to demographic and personality dimensions than to current emotional well-being. These findings emphasize the multifactorial nature of BR decision-making in the preoperative setting.

## 4. Discussion

This study examined the demographic, psychosocial, and personality-related factors influencing women’s preoperative decision-making regarding breast reconstruction (BR) following mastectomy. By focusing on the preoperative phase, it offers valuable insights into the psychological and contextual variables that shape both BR intention and information-seeking behavior—an area that remains underexplored. Conducted in a regional academic hospital in northern Greece, the study contributes evidence from a setting typically underrepresented in BR research, thereby enhancing the diversity and applicability of findings across healthcare contexts.

Demographic characteristics, particularly age and marital status, emerged as meaningful predictors. Consistent with previous research, older women were significantly less likely to express BR intention or seek related information. This trend reflects broader patterns in the literature, where BR uptake among women over 65 remains disproportionately low despite high incidence rates of breast cancer in this age group. Potential explanations include increased concern about surgical risks, comorbidities, and reduced emphasis on body image. Conversely, younger women have been found to prioritize appearance-related outcomes and psychosocial recovery more often, making BR a more salient consideration [23].

Marital status was also associated with information-seeking behavior. Married women were more likely to engage with BR-related information than those who were single or divorced. This pattern aligns with findings indicating that emotional and logistical support from a partner facilitates decision-making and reduces uncertainty [13,38]. Such support may foster greater engagement with healthcare information and more confident treatment planning. For patients without close support networks, supplementary interventions—such as peer-led counseling or caregiver involvement—may be particularly beneficial.

Health-related quality of life (HRQoL) emerged as a significant psychological correlate of BR intention. Specifically, women reporting higher physical functioning were more likely to express intent to pursue BR, suggesting that perceived physical readiness may enhance willingness to engage in additional surgical interventions [25]. Conversely, higher scores on the SF-36 Mental Component Summary (MCS)—which reflects greater emotional well-being—were associated with reduced BR intent. Although initially counterintuitive, this finding aligns with studies indicating that individuals with greater psychological stability and emotional resources may experience higher body acceptance and feel less compelled to pursue reconstruction for emotional adjustment [39,40,41]. The SF-36 MCS captures positive emotional states, including vitality and role emotional functioning, and has been linked to adaptive coping and lower emotional burden in women with breast cancer [39]. Thus, women with stronger emotional adjustment may feel more reconciled with mastectomy outcomes and perceive less personal benefit from BR.

In contrast, depressive symptomatology, as measured by HADS-depression, was also negatively associated with BR intention, but likely reflects a distinct psychological pathway. Women with elevated depression scores were less inclined to consider BR, consistent with evidence that depressive affect can impair motivation and decisional engagement in elective healthcare contexts [42,43]. This association is conceptually distinct from the SF-36 MCS findings. While higher MCS scores reflect emotional well-being and psychological adjustment, elevated HADS-depression scores indicate active emotional distress. Together, these results suggest that both emotional well-being and emotional burden can reduce BR intent, albeit through different mechanisms: one through acceptance, the other through demotivation or psychological disengagement. This dual explanation aligns with prior literature [39,40,41,42,43].

Interestingly, neither anxiety nor depressive symptoms were significantly associated with the desire to receive BR-related information. This suggests that psychological distress may exert a more pronounced influence on decisional readiness than their interest in acquiring information. Similarly, HRQoL was not significantly linked to information-seeking behavior, implying that such behavior may be shaped more by dispositional or contextual variables—such as personality traits, education level, or social support—rather than perceived health status. Additionally, it is possible that emotional or cognitive overload among distressed patients may lead to avoidance of health information, as observed in prior decision-making research [15,19]. These complementary findings underscore the need for comprehensive preoperative assessment of both emotional burden and psychological preparedness as key dimensions of patient-centered BR counseling [22,23,24].

Among the personality traits examined, only openness to experience was significantly associated with both BR intention and information-seeking. Women scoring higher in openness—a trait associated with curiosity, adaptability, and cognitive flexibility—were more likely to consider BR and actively seek related information. These results are in line with prior studies linking openness to health-related information engagement and shared decision-making [16,20]. However, neuroticism and extraversion did not show significant associations in this sample, despite their documented relevance in other surgical populations [18]. It is possible that cognitive-exploratory traits, such as openness, exert a stronger influence during the preoperative phase, when patients are still forming preferences, while affective traits such as neuroticism may play a more significant role in postoperative adjustment.

These findings carry important implications for multidisciplinary breast cancer care and preoperative planning. First, the observed age-related disparities in BR engagement underscore the value of tailoring educational strategies to the specific priorities and concerns of older patients. This may involve more nuanced discussions around comorbid conditions, surgical risk–benefit considerations, and the psychosocial value of BR at later stages of life [8,11]. This is consistent with our recent systematic review, which identified age, emotional readiness, and informational clarity as key factors influencing BR decision-making across diverse clinical contexts [31]. Second, the influence of marital status highlights the need to assess patients’ available support systems during surgical consultations. For women without partners, integrating peer-led support, caregiver involvement, or referrals to psychosocial services may help mitigate decisional burden and foster greater autonomy [1,28]. Third, the nuanced role of mental health—particularly the dual influence of psychological distress and resilience—suggests that routine psychosocial screening should be embedded in pre-surgical evaluations to better identify those requiring targeted support [4,22,23,24]. Finally, acknowledging individual differences in personality traits may enhance patient–clinician communication; integrating personality-informed counseling approaches could foster more meaningful engagement and better alignment between medical recommendations and patient preferences [15,20].

Taken together, these findings support a comprehensive model of preoperative care that includes demographic profiling, psychological assessment, personality evaluation, and HRQoL screening. Such an approach can help identify women who may benefit from tailored mental health promotion strategies and ensure that BR decisions are informed, individualized, and emotionally supported.

### Study Limitations

Several methodological considerations should be acknowledged when interpreting the findings of this study. The relatively small sample size may have limited the statistical power needed to detect subtle associations and precluded the use of multivariable models capable of accounting for interaction effects. Consequently, the reported associations should be interpreted as exploratory and hypothesis-generating rather than conclusive. Additionally, as the study was conducted at a single academic center in Greece, in a regional city distant from the country’s major urban centers—its findings may not reflect the experiences of patients in more metropolitan healthcare settings. Cultural and regional variations in healthcare delivery, surgical practices, and attitudes toward BR could affect the applicability of these results in other contexts. Nevertheless, this context offers valuable insight into the decision-making processes of women treated outside large urban hospitals, contributing to the evidence based on BR preferences among geographically and potentially socioeconomically diverse populations. The cross-sectional design, which relied exclusively on preoperative data, also limits the ability to draw causal inferences or assess changes over time. A longitudinal approach is needed to determine whether the observed predictors persist and ultimately influence surgical outcomes. Moreover, all psychological, personality, and quality of life variables were self-reported, which may introduce response bias despite the use of standardized and validated instruments. Finally, the study did not evaluate broader structural determinants, such as socioeconomic status, health literacy, or healthcare accessibility, all of which are known to shape decision-making and access to BR. Future research should incorporate detailed measures of socioeconomic status, health literacy, and access to BR services—such as travel distance to surgical centers, waiting times, and financial coverage—so as to better understand how structural factors influence both the intention to undergo BR and the ability to act on that intention.

## 5. Conclusions

This study offers valuable insights into the psychological, personality, and demographic factors that shape women’s preoperative decision-making about BR following a breast cancer diagnosis. Age, marital status, openness to experience, and perceived quality of life—particularly in physical and mental health domains—emerged as significant influences on both the desire for BR-related information and the intention to pursue BR. Although psychological distress did not predict information-seeking behavior, it was associated with reduced BR intent, underscoring the critical role of emotional well-being in treatment decision-making.

These findings highlight the need for a comprehensive, mental health–informed, person-centered approach to preoperative counseling. Integrating psychosocial screening, personality-informed communication strategies, and systematic assessment of quality of life can enhance decision support, particularly for women vulnerable to emotional distress during the cancer treatment process. By proactively addressing psychosocial needs, the multidisciplinary breast cancer care team can play a pivotal role in promoting mental health, fostering emotional resilience, and supporting informed, individualized surgical decisions. This mental health-promoting framework holds promise for improving outcomes and reducing disparities among women undergoing mastectomy.

## Figures and Tables

**Table 2:** Results of binary logistic regression analysis for the effect of demographic characteristics on the desire for BR information.

Demographic Characteristics		b	*p*	Exp(b)	Confidence Interval for Exp(b)	
					Lower	Upper
Religion group	1 = Christian	0.299	0.737	1.349	0.236	7.718
	2 = Muslim	0		1		
Residence	1 = Urban area	−0.778	0.232	0.460	0.129	1.643
	2 = Non-urban area	0		1		
Professional status	1 = Unemployed	0.136	0.893	1.145	0.159	8.276
	2 = Employed	−0.242	0.808	0.785	0.112	5.490
	3 = Retired	0		1		
Age		−0.078	**0.041**	**0.925**	0.859	0.997
Education_level		0.145	0.136	1.156	0.955	1.398
Marital status	1 = Single/divorced	−2.935	**0.014**	**0.053**	0.005	0.549
	2 = Widowed	0.398	0.588	1.488	0.353	6.265
	3 = Married	0		1		

Abbreviations: b = Unstandardized regression coefficient, *p* = *p*-value (probability), and Exp(b) = odds ratio. Source: own elaboration. Note: values in bold indicate statistically significant results (*p* < 0.05 or 95% CI not including 1).

**Table 3:** Results of binary logistic regression analysis for the effect of demographic characteristics on the intention to undergo BR.

Demographic Characteristics		b	*p*	Exp(b)	Confidence Interval for Exp(b)	
					Lower	Upper
Religion group	1 = Christian	0.808	0.367	2.243	0.388	12.955
	2 = Muslim	0		1		
Residence	1 = Urban area	−0.165	0.798	0.848	0.240	2.993
	2 = Non-urban area	0		1		
Professional status	1 = Unemployed	−0.446	0.644	0.64	0.097	4.238
	2 = Employed	−0.716	0.472	0.489	0.069	3.440
	3 = Retired	0		1		
Age		−0.109	**0.007**	**0.897**	0.829	0.970
Education_level		0.074	0.393	1.077	0.909	1.275
Marital status	1 = Single/divorced	−0.905	0.258	0.404	0.084	1.939
	2 = Widowed	−0.588	0.427	0.556	−0.130	2.367
	3 = Married	0		1	0.388	12.955

Abbreviations: b = Unstandardized regression coefficient, *p* = *p*-value (probability), and Exp(b) = odds ratio. Source: own elaboration. Note: values in bold indicate statistically significant results (*p* < 0.05 or 95% CI not including 1).

**Table 4:** Results of binary logistic regression analysis for the effect of personality characteristics on the desire for BR information.

IPIP-BFFM Parameter	b	*p*	Exp(b)	Confidence Interval for Exp(b)	
				Lower	Upper
IPIP_Extraversion	−0.017	0.679	0.984	0.909	1.064
IPIP_Agreeableness	0.049	0.422	1.051	0.931	1.185
IPIP_Conscientiousness	−0.090	0.086	0.914	0.825	1.013
IPIP_Neuroticism	0.026	0.377	1.026	0.969	1.087
IPIP_Openness	0.109	**0.009**	**1.115**	1.028	1.209

Abbreviations: IPIP-BFFM = International Personality Item Pool Big-Five Factor Model, b = unstandardized regression coefficient, *p* = *p*-value (probability), and Exp(b) = odds ratio. Source: own elaboration. Note: values in bold indicate statistically significant results (*p* < 0.05 or 95% CI not including 1).

**Table 5:** Results of binary logistic regression analysis for the effect of personality characteristics on the intention to undergo BR.

IPIP-BFFM Parameter	b	*p*	Exp(b)	Confidence Interval for Exp(b)	
				Lower	Upper
IPIP_Extraversion	0.007	0.851	1.007	0.935	1.086
IPIP_Agreeableness	0.007	0.907	1.007	0.897	1.130
IPIP_Conscientiousness	−0.013	0.797	0.987	0.895	1.089
IPIP_Neuroticism	0.030	0.290	1.031	0.974	1.091
IPIP_Openness	0.091	**0.017**	**1.095**	1.016	1.181

Abbreviations: IPIP-BFFM = International Personality Item Pool Big-Five Factor Model, b = unstandardized regression coefficient, *p* = *p*-value (probability), and Exp(b) = odds ratio. Source: own elaboration. Note: values in bold indicate statistically significant results (*p* < 0.05 or 95% CI not including 1).

**Table 6:** Results of binary logistic regression analysis for the effect of psychological characteristics on the desire for BR information.

Parameter	b	*p*	Exp(b)	Confidence Interval for Exp(b)	
				Lower	Upper
SF36_ Physical health	0.014	0.324	1.014	0.987	1.041
SF36_ Mental health	−0.02	0.284	0.98	0.945	1.017
HADS_Anxiety	−0.044	0.545	0.957	0.831	1.103
HADS_Depression	−0.085	0.399	0.918	0.753	1.12

Abbreviations: SF36 = 36-Item Short Form Survey, HADS = Hospital Anxiety Depression Scale, b = unstandardized regression coefficient, *p* = *p*-value (probability), and Exp(b) = odds ratio. Source: own elaboration.

**Table 7:** Results of binary logistic regression analysis for the effect of psychological characteristics on the intention to undergo BR.

Parameter	b	*p*	Exp(b)	Confidence Interval for Exp(b)	
				Lower	Upper
SF36_Physical health	0.039	**0.016**	**1.039**	1.007	1.072
SF36_Mental health	−0.049	**0.025**	**0.952**	0.912	0.994
HADS_Anxiety	0.01	0.895	1.01	0.872	1.169
HADS_Depression	−0.226	**0.046**	**0.797**	0.638	0.996

Abbreviations: SF36 = 36-Item Short Form Survey, HADS = Hospital Anxiety Depression Scale, b = unstandardized regression coefficient, *p* = *p*-value (probability), and Exp(b) = odds ratio. Source: own elaboration. Note: Values in bold indicate statistically significant results (*p* < 0.05 or 95% CI not including 1).

## Data Availability

The data supporting the findings of this study are available from the corresponding author upon reasonable request. Access is restricted due to ethical and privacy considerations.

## Abbreviations

The following abbreviations are used in this manuscript:

BR	Breast Reconstruction
IPIP-BFFM	International Personality Item Pool-Big Five-Factor Markers
HADS	Hospital Anxiety Depression Scale
SF-36	36-Item Short Form Survey
QoL	Quality of Life
DIEP	Deep Inferior Epigastric Perforator
PCS	Physical Component Summary
MCS	Mental Component Summary
SD	Standard Deviation
IBM	International Business Machines Corporation
SPSS	Statistical Package for the Social Sciences
HRQoL	Health-Related Quality of Life
